# Loss of uterine EGR2 contributes to age-associated decline in fertility in female mice.

**DOI:** 10.17912/micropub.biology.001852

**Published:** 2025-11-13

**Authors:** Sudikshya Paudel, Magdalina J. Cummings, Steven L. Young, Xiaoqiu Wang

**Affiliations:** 1 Department of Animal Science, North Carolina State University, Raleigh, North Carolina, United States; 2 Reproductive Endocrinology and Infertility, Obstetrics and Gynecology, Duke University School of Medicine, Durham, North Carolina, United States; 3 The Comparative Medicine Institute, North Carolina State University, Raleigh, North Carolina, United States

## Abstract

Early growth response 2 (Egr2) is a pleiotropic zinc finger transcription factor with established roles in neural and immune system, but its uterine function remains poorly understood. We found that uterine EGR2 is expressed in luminal epithelium, glandular epithelium, and stroma of human and mouse uteri, with dynamic regulation across the menstrual cycle and early pregnancy. EGR2 expression declined in aged and *Sirt1* -deficient mouse uteri, models of reproductive aging. Conditional uterine deletion of EGR2 caused mild subfertility, with fewer litters and total pups per female. These findings indicate EGR2 supports, but is not essential for, uterine function.

**Figure 1. Uterine Egr2 expression is dynamically regulated, and its loss causes mild subfertility in female mice.**
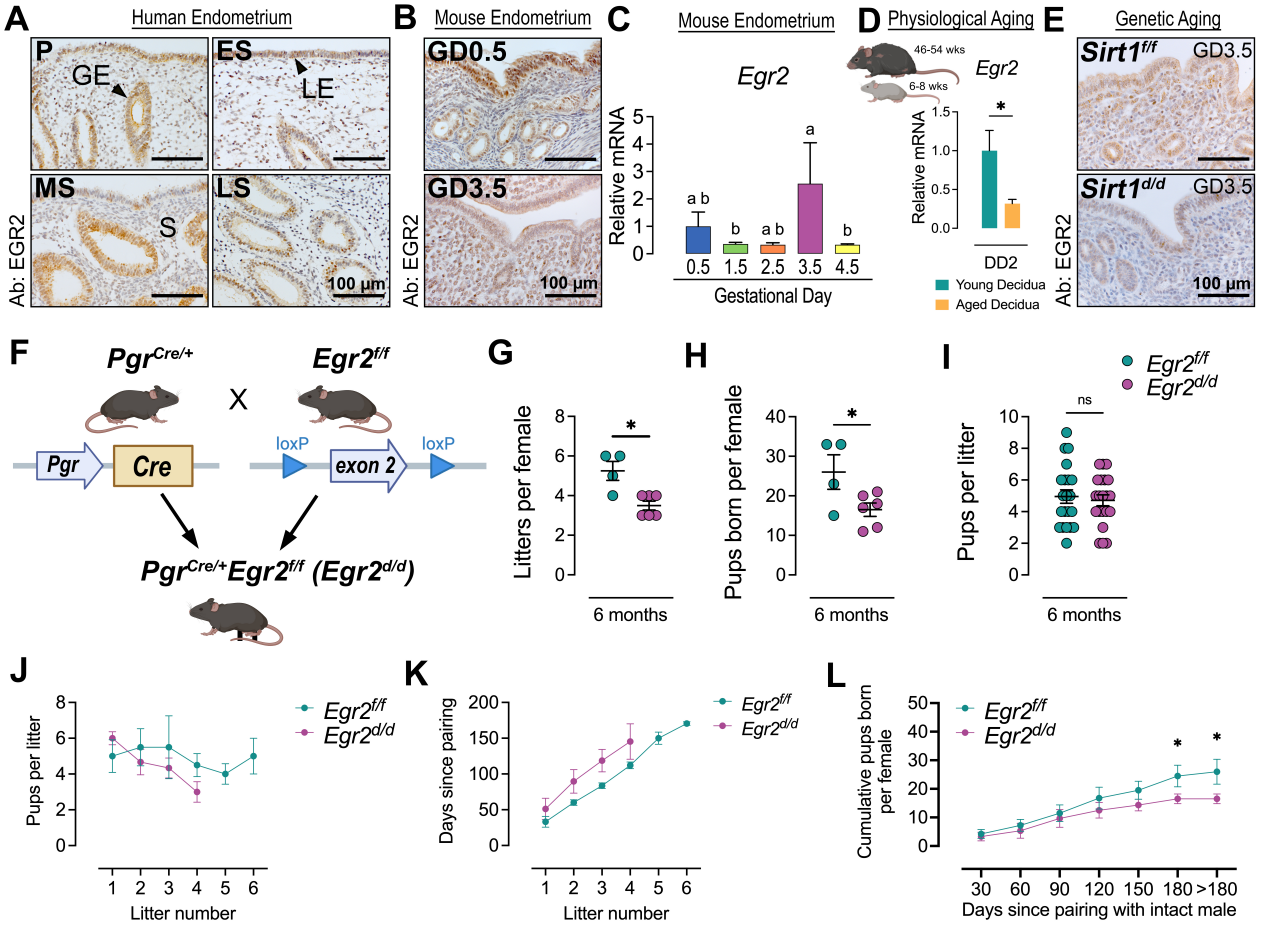
**(A** ) Immunohistochemistry (IHC) of human endometrium showing EGR2 protein in luminal epithelium (LE), glandular epithelium (GE), and stroma (S) across the menstrual cycle. EGR2 increased in GE at the mid-secretory (MS) phase and decreased at the late secretory (LS) phase. P, proliferative; ES, early secretory. Scale bar, 100 mm. **(B)** IHC of wild-type C57BL/6J mouse uteri showing EGR2 in LE, GE, and stroma, with expression elevated between gestational day (GD) 0.5 and GD 3.5. Scale bar, 100 mm. **(C)** qRT-PCR quantification of uterine *Egr2* mRNA from GD 0.5-4.5, demonstrating reduced expression between GD 0.5 and 2.5, a peak at GD 3.5, and downregulation at GD 4.5. Different superscript letters denote significant differences ( *P* < 0.05, one-way ANOVA with Fisher’s LSD test). **(D)** qRT-PCR analysis of uterine decidua at decidual day 2 (DD2, equivalent to GD 6.5) showed reduced *Egr2* in aged (46-54 weeks of age) versus young (6-8 weeks of age) females following *in vivo* artificial decidualization. * *P* < 0.05 (two-tailed Student’s t test). **(E)** IHC of uteri from *Pgr ^Cre/+^ Sirt1 ^f/f^* ( *Sirt1 ^d/d^* ) mice at GD 3.5 showed reduced EGR2 in LE, GE, and stroma compared with *Sirt1 ^f/f^* controls. **(F)** A schematic illustrated the breeding strategy for generating conditional uterine *Egr2* knockout mice using Pgr-Cre and floxed Egr2 (exon 2 flanked). **(G-I)** Fertility of *Egr2 ^f/f^* (control) and *Egr2 ^d/d^* females was assessed over 6 months. (G) Litters numbers per female, (H) total pups per female, and (I) pups per litter were recorded. Each dot represented one animal (G, H) or one litter (I). * *P* < 0.05 (two-tailed Student’s t test). **(J-L)** Reproductive performance of *Egr2 ^f/f^* and *Egr2 ^d/d^* females was further evaluated. (J) Litter number distribution, (K) time taken to produce a specific number of litters, and (L) cumulative pups since pairing are shown. **(J)** Pups born in a specific number of litters. **(K)** Time taken to produce a specific number of litters. **(L)** Cumulative pups born since pairing. Data are presented as mean ± SEM; >180 days denoted the final count of pups. * *P* <0.05 (two-way ANOVA with Tukey’s multiple comparisons test) at specific time points for contrast between *Egr2 ^f/f^* (green, n=4) and *Egr2 ^d/d^* (purple, n=6).

## Description

Early growth response 2 (EGR2, also known as KROX20), is a zinc finger transcription factor that regulates diverse biological processes, including hindbrain development and T cell differentiation (De and Turman, 2005; Kim et al., 2015). As a member of the EGR family (EGR1-4), EGR2 acts as an immediate-early gene induced by extracellular cues such as growth factors, hormones, and cytokines (O'Donovan et al., 1999). EGR2 has been studied extensively in the nervous and immune systems, where it is indispensable for Schwann cell differentiation, myelination, and peripheral tolerance (Topilko et al., 1994; Warner et al., 1998; Safford et al., 2005). However, its role in reproductive physiology remains poorly understood. In the female reproductive tract, uterine receptivity and blastocyst implantation are orchestrated by dynamic transcriptional networks integrating ovarian steroid hormones with paracrine factors from the endometrium and conceptuses (Cha et al., 2012; Wang et al., 2017). Within this context, EGR1 is well characterized, with established functions in gonadotropin signaling (Lee et al., 1996) and endometrial stromal cell decidualization (Szwarc et al., 2019; Maurya et al., 2022; Maurya et al., 2023). Far less is known about EGR2 in the uterus. Emerging evidence suggests that EGR2 may contribute to uterine function through multiple pathways. In mice, EGR2 mediates stromal cell decidualization downstream of HB-EGF stimulation (Liu et al., 2017; Yue et al., 2018), while in human endometrial stromal cells it is directly regulated by promyelocytic leukemia zinc finger (PLZF), a progesterone receptor target (Szwarc et al., 2018). We previously reported that uterine *Egr2* mRNA expression is reduced in mouse models with epithelial SOX17 deficiency (Wang et al., 2018) and in SIRT1-deficient uteri, which represent a model of premature reproductive aging (Cummings et al., 2022). Collectively, these findings implicate EGR2 as a potential modulator of epithelial-stromal communication, endometrial remodeling.

In this study, we examined EGR2 expression in human endometrium across the menstrual cycle. Immunohistochemistry (IHC) revealed that EGR2 protein in luminal epithelium (LE), glandular epithelium (GE), and stroma throughout the menstrual cycle ( **Fig. 1A** ). Expression was detectable in both epithelial and stromal compartments during the proliferative and early secretory phases, became more prominent in GE during the mid-secretory phase, and declined in the late secretory phase. These findings suggest that EGR2 is dynamically regulated during the menstrual cycle and may contribute to the establishment of uterine receptivity, particularly during the mid-secretory “window of implantation”. Consistent with human data, EGR2 was present in LE, GE, and stroma of wild-type mouse uteri, with increased expression between gestation day (GD) 0.5 and GD 3.5 ( **Fig. 1B** ). The qRT-PCR confirmed dynamic regulation, with *Egr2* mRNA levels declining between GD 0.5 and GD 2.5, peaking at GD 3.5, and decreasing again by GD 4.5 ( **Fig. 1C** ). This temporal expression pattern aligns with events critical for uterine receptivity and conceptus attachment.

We next examined the relationship between EGR2 and reproductive aging. Uterine decidua from aged female mice (46-54 weeks of age) showed reduced *Egr2* mRNA expression compared with young females (6-8 weeks of age) following artificial decidualization ( **Fig. 1D** ). Similarly, diminished EGR2 protein was observed in LE, GE, and stroma of *Sirt1 ^d/d^* mice, a model of premature uterine aging (Cummings et al., 2022), at GD 3.5 relative to *Sirt1 ^f/f^* controls ( **Fig. 1E** ). These results indicate that EGR2 expression is sensitive to reproductive aging and SIRT1-dependent pathways, suggesting it may participate in age-associated changes in uterine function.

To assess the functional role of uterine EGR2, we generated conditional knockout mouse model, *Pgr ^Cre/+^ Egr2 ^f/f^* ( *Egr2 ^d/d^* ; **Fig. 1F** ). In a 6-month breeding trial, *Egr2 ^d/d^* females produced fewer litters per female ( **Fig. 1G** ) and reduced total pups per female ( **Fig. 1H, 1L** ) compared with *Egr2 ^f/f^* controls. However, litter size ( **Fig. 1I, 1J** ) was unaffected. Mating efficiency was comparable across genotypes because all females were bred with wildtype C57BL/6J males of proven fertility and no difference in inter-litter interval was observed ( **Fig. 1K** ). Thus, loss of uterine EGR2 causes only mild subfertility, reflected in reduced overall reproductive output but not in litter size or timing. We acknowledge that the overall sample sizes were relatively small (n=4-6 per genotype), which may limit statistical power and generalizability. However, the selected sample sizes were based on prior power analysis and extensive experimental experience (Wang et al., 2018; Cummings et al., 2022), and were sufficient to detect biologically meaningful differences under our experimental conditions.

In conclusion, our study shows that uterine EGR2 expression is dynamically regulated during the menstrual cycle and early pregnancy, declines with reproductive aging, and its loss modestly impairs reproductive capacity. These findings suggest that EGR2 contributes to, but is not essential for, uterine function and fertility. 

## Methods

**Human endometrial samples**

This study was carried out in accordance with federal regulations for human subjects research. All procedures were approved by the Institutional Review Boards of Duke University School of Medicine, and informed consent was obtained from all participants prior to enrollment. A total of 12 samples were analyzed, representing the proliferative, early secretory, mid-secretory, and late secretory phases of the menstrual cycle (n=3 per phase).

**Animals**

All experimental procedures involving animals were approved by the Animal Care and Use Committee of North Carolina State University and performed in accordance with its guidelines. C57BL/6J mice were used throughout this study. ‘Young’ virgin females were generally 6–8weeks old and ‘aged’ virgin females were 46–54 weeks old. Timed matings were set up with standard C57BL/6J males between 8 and 16 weeks of age with proven fertility. The morning when the vaginal plug was detected was designate gestational day (GD) 0.5. The uterine-specific knockout mice ( *Sirt1 ^d/d^ ; Egr2 ^d/d^* ) were generated by crossing *Pgr ^Cre/^* ^+^ mice (Soyal et al., 2005) with mice carrying *Sirt1 ^f/f^* (Cummings et al., 2022) or *Egr2 ^f/f^* alleles (Taillebourg et al., 2002), respectively. Conditional knockout and control littermate females at 6 weeks of age were housed individually and continuously with wildtype C57BL/6J males of proven fertility. Mating was confirmed by the presence of vaginal plugs. Fertility was assessed by monitoring the frequency of litters and litter sizes for a 6-month period.

**Artificial decidualization**

To assess the ability of the stroma to undergo differentiation and proliferation independent of implantation by a blastocyst, aged or young female mice were ovariectomized and treated with exogenous hormones to mimic pregnancy before applying a manual stimulus to a single uterine horn (Finn and Martin, 1972). After ovariectomy and 2 weeks of rest to eliminate endogenous ovarian steroids, mice were administered E2 (E8875, Sigma-Aldrich, St. Louis, MO, USA; 100 ng per mouse) as daily injections for 3 days. After 2 days of rest, mice were treated with E2 (6.7 ng per mouse) and P4 (P0130, Sigma-Aldrich; 1 mg per mouse) for 3 days. On the third day (DD0), mice were administered a single injection of 0.05 ml sesame oil into the right uterine horn. Mice were administered E2 and P4 for 2 more days and euthanized on the second day (DD2). Uterine wet weights for the stimulated and control horns were recorded. Weight ratios were calculated by dividing stimulated horn weight by unstimulated horn weight. The whole decidual horn was used in the following analyses.

**Immunohistochemistry**

Uterine tissue was fixed in 4% v/v PFA and embedded in paraffin wax. Embedded tissues were sectioned at 5 mm and baked 1h at 60°C. After cooling, slides were dewaxed using Citrisolv clearing agent (22-143-975, Thermo Fisher) through a decreasing gradient of pure ethanol. Antigen retrieval was performed according to the manufacturer’s instructions using Antigen Unmasking Solution (H-3300, Vector Labs). Endogenous peroxidase activity was quenched with 3% hydrogen peroxide in methanol. Sections were blocked with 5–10% normal donkey serum before incubation with the primary EGR2 antibody (P100880-P050, AVIVA Systems Biology) overnight at 4°C. When required, the secondary antibody was diluted in 1:200 in 1% bovine serum albumin. The ABC reagent (PK-6100, Vector Labs) was applied according to the manufacturer’s instructions. Signal was developed using DAB ImmPACT staining (SK-4105, Vector Labs), followed by hematoxylin counterstaining. Slides were dehydrated and coverslipped for imaging.

**RNA isolation and quantitative real-time PCR analyses**

Frozen tissue was homogenized in 1 ml of TRIzol reagent (Thermo Fisher) using a Bead Mill 24 homogenizer (Thermo Fisher), two times at 4.5 m/s for 30 s, and rested intermittently for 20 s on ice. Homogenates were centrifuged for 10 min at 12,500 *g* at 4°C to pellet cellular debris. The supernatant was transferred to a 1.5-ml centrifuge tube and mixed with 200 ml of 1-bromo-3-chloropropane by manually shaking for 20 sec. The tube was then incubated at room temperature for 3 min, and centrifuged for 18 min at 12,500 *g* at 4°C. The upper aqueous phase (~400 ml) was carefully removed, placed into a new 1.5-ml tube, and mixed with 200 ml of chloroform by shaking the tubes for 20 sec. Samples were rested for 3 min at room temperature and subsequently centrifuged at 21,000 *g* for 18 min at 4°C. Approximately 500 ml of the aqueous layer was transferred to a new tube and mixed with equal parts of 70% ethanol. This mix was filtered in columns from the RNeasy Mini kit (Qiagen, Valenica, CA, USA). Columns were washed once with 700 ml of RW1 buffer (1053394; Qiagen) and three times with 500 ml of RPE buffer (1018013; Qiagen). The RNA was eluted with 30 ml RNase-free water. The quantity and quality of total RNA were determined using spectrometry and denaturing agarose gel electrophoresis, respectively. RNA was reverse transcribed into cDNA using the M-MLV Reverse Transcriptase (28025021; Thermo Fisher) according to the manufacturer’s instructions. Quantitative RT-PCR (qRT-PCR) was performed using the CFX Connect Real-Time PCR Detection System (Bio-Rad, Hercules, CA, USA) and the SsoAdvanced™ Universal SYBR® Green Supermix (1725274; Bio-Rad) with oligonucleotide primers 5’-CTCCCGTATCCGAGTAGC-3’ ( *Egr2* forward), 5’-GATGCCCGCACTCACAAT-3’ ( *Egr2* reverse) synthesized by Integrated DNA Technologies (IDT; Coralville, IA, USA). Delta delta Ct values were calculated using 18 S ribosomal RNA control amplification results to determine relative variations in expression of mRNAs per sample.

**Statistical analyses**

Normality of data and homogeneity of variance were tested using the Shapiro-Wilk test and Brown-Forsythe test in Statistical Analysis System, respectively (version 8.1; SAS Institute). Data were analyzed by least squares one-way analysis of variance (ANOVA) and post hoc analysis (the Fisher least significant difference) with each mouse as an experimental unit. All analyses were performed using SAS and visualized with GraphPad Prism software (version 10.6.1). Data are expressed as means with SEM, and differences were considered statistically significant at *P* <0.05.
